# The Resistance Mechanisms of Lung Cancer Immunotherapy

**DOI:** 10.3389/fonc.2020.568059

**Published:** 2020-10-20

**Authors:** Fen Wang, Shubin Wang, Qing Zhou

**Affiliations:** ^1^Guangdong Provincial Key Laboratory of Translational Medicine in Lung Cancer, Guangdong Provincial People’s Hospital, Guangdong Academy of Medical Sciences, School of Medicine, Guangdong Lung Cancer Institute, South China University of Technology, Guangzhou, China; ^2^Shenzhen Key Laboratory of Gastrointestinal Cancer Translational Research, Department of Oncology, Cancer Institute of Shenzhen-PKU-HKUST Medical Center, Peking University Shenzhen Hospital, Shenzhen, China

**Keywords:** resistance mechanism, immunotherapy, PD-1/PD-L1, Immune check inhibitor, lung cance

## Abstract

Immunotherapy has revolutionized lung cancer treatment in the past decade. By reactivating the host’s immune system, immunotherapy significantly prolongs survival in some advanced lung cancer patients. However, resistance to immunotherapy is frequent, which manifests as a lack of initial response or clinical benefit to therapy (primary resistance) or tumor progression after the initial period of response (acquired resistance). Overcoming immunotherapy resistance is challenging owing to the complex and dynamic interplay among malignant cells and the defense system. This review aims to discuss the mechanisms that drive immunotherapy resistance and the innovative strategies implemented to overcome it in lung cancer.

## Introduction

The discovery of the immune checkpoint inhibitors (ICIs), represented by the monoclonal antibodies that block cytotoxic T−lymphocyte−associated protein 4 (CTLA-4), programmed death protein 1 (PD-1), and programmed death protein ligand 1 (PD-L1), has revolutionized the therapeutic landscape of lung cancer. The significant survival benefit derived from ICI-containing treatment has established it as the mainstay first-line therapy in patients with advanced or locally advanced non-small cell lung cancer (NSCLC) and extensive small-cell lung cancer (SCLC). Unprecedented long-term clinical benefit or even, in some cases, a complete recovery has been witnessed in lung cancer, particularly in patients with high PD-L1-expressing tumors (1–3). Currently, investigations are under way aimed at integrating immunotherapy in the treatment of early-stage lung cancer.

However, most patients with NSCLC develop primary resistance during ICI monotherapy and only 15 to 20% achieve partial or complete response (3). Acquired resistance also occurs in initially responding patients with advanced NSCLC treated with ICIs, after a median progression-free survival (PFS) of 4–10 months (4–9). The mechanisms of resistance to immunotherapy are not yet fully understood, and methods to overcome them must be developed. Herein, we discuss the pathways driving resistance to immunotherapy in lung cancer to help clinicians in their current practice, as well as identify future research priorities and treatment strategies.

## Different Schemas of Resistance to Immunotherapy

Unlike molecular targeted therapy and chemotherapy targeting tumor cells, immunotherapy targets the immune system of the host by mobilizing the immune cells to recognize and eventually eliminate tumor cells. This mechanism of action determines the complexity of the resistance mechanisms in immunotherapy. Different mechanisms of immunotherapy resistance are listed in Table 1.

**TABLE 1:** Different schemas of resistance to immunotherapy.

Schemas	Classifications	Description
Temporal perspective	Primary	Lack of initial response or clinical benefit to therapy
	Acquired	Disease progression after an initial period (6 months) of clinical benefit
Spatial perspective	Intrinsic	Tumor-related resistance
	Extrinsic	Factors involved in microenvironment or tumor-immunity cycle
Immunological perspective	Immune inflamed	Tumor inhibits immune activities notwithstanding abundant immune cells infiltration
	Immune desert	Tumor fails to evoke an immunoreaction
	Immune excluded	Tumor prevents immune cells infiltration in spite of adequate immunogenicity

In accordance with the timing of development, resistance can be considered as either primary, when no initial response or clinical benefit to the therapy is observed, or acquired, as disease progression occurs after an initial period of clinical benefit (10). Clinically, 6-month treatment duration is adopted as a cutoff value (11). This classification schema correlates with real-time observations by clinicians and contributes to the clinical decision-making process in the absence of other information such as immune characteristics and tumor genetics.

Resistance is additionally classified as intrinsic or extrinsic to cancer cells. The former occurs in the tumor cell itself and encompasses the inherent characteristics related to gene expression, cell signaling, immune recognition, and DNA damage response, whereas the latter is seen in the microenvironment or systemic circulation throughout the T-cell bioactivation process (12, 13).

The cancer−immunity cycle is linked to immunotherapy resistance in another related schema (14). This classification divides resistance from an immunological perspective into immune desert (tumor fails to evoke an immune reaction), immune inflamed (tumor inhibits immune activities notwithstanding abundant immune cells infiltration), or excluded (tumor prevents immune cells infiltration in spite of adequate immunogenicity) (13).

It is noteworthy that the immune response is a continuous and dynamic process rather than categorical (binary). Multiple complex interactions, including immunologic, genomic, and host characteristics and treatment interventions, rather than a single, dominant determinant are involved in the resistance to immunotherapy. The fs can be overlapping or parallel in some cases despite the different timing of occurrence (11).

## Resistance Mechanisms to Immunotherapy

**FIGURE 1:**
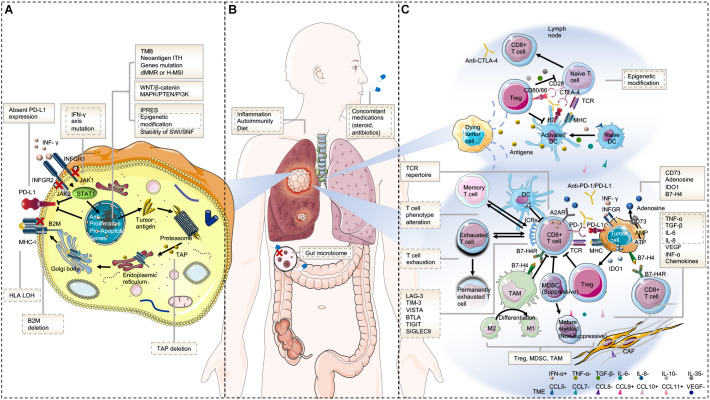
Mechanisms of resistance to immunotherapy. **(A)** Tumor intrinsic mechanisms that are associated with resistance to immunotherapy include lack of tumor immunogenicity (low TMB, heterogenous antigens, mutation of certain genes, and IPRES transcriptional signatures), deficiency in antigen presentation (alterations in INF-γ signaling pathway, HLA LOH, B2M, and TAP deletion), aberrations in several signaling pathways (MAPK, PI3K, WNT, and IFN), and absent PD-L1 expression. **(B)** Host-related characteristics that lead to primary or secondary resistance include the gut microbiome, diet, concomitant medications, inflammation state, and autoimmunity. **(C)** Tumor extrinsic mechanisms involved in resistance to immunotherapy include T cell-related factors (alternative immune checkpoints, T cell exhaustion and phenotype alteration, TCR repertoire, and epigenetic modification), immunosuppressive cells (Treg, MDSC, and M2-TAM), and cytokines and metabolites (e.g., TGF-β, adenosine) released into the tumor microenvironment. Factors in the solid text boxes are involved in primary resistance, whereas those in the dotted text boxes are involved in secondary or acquired resistance. Factors with solid and dotted dual text boxes are involved in both. Cytokines with “+” and “-” represent positive and negative modulators to antitumor immune response, respectively. Abbreviations: TME, tumor microenvironment; MHC, major histocompatibility complex; TCR, T cell receptor; Treg, regulatory T cell; MDSC, myeloid-derived suppressor cell; M2-TAM, type II tumor-associated macrophage; ICR, immune checkpoint receptor; CAF, cancer-associated fibroblast; and IPRES, innate anti-PD-1 resistance.

Underlying mechanisms of primary resistance span an extensive range from tumor factors including genomic features, transcriptomic signatures, and immune landscape, to host factors. The potential mechanisms of acquired resistance at least partly overlap with those involved in primary resistance and mainly include loss of neoantigen and deficiency in presentation, loss of T-cell effector function, and up-regulation of alternate immune checkpoint receptors (10). Here, we will discuss the mechanisms of resistance to immunotherapy from tumor aspects (intrinsic and extrinsic mechanisms) and host-related characteristics in order to avoid confusion and repetition (Figure 1).

### Tumor Cell-Intrinsic Mechanisms

#### Genomic Features

##### Low tumor mutation burden and neoantigen load

Tumor-specific antigens are the key to activate T cells to recognize tumor as foreign, which is the first step of tumor-induced adaptive immune responses and immune-mediated tumor killing (15). These neoantigens, interestingly, are derived from somatic mutations and contain new epitopes, and subsequently lead to tumor immunogenicity. Preclinical and clinical studies have revealed that the response of neoantigen-specific effector T cell (Teff) paralleled tumor shrinkage (16–20).

With the improvement of sequencing techniques, it was found that nonsynonymous mutations can generate neoantigens that trigger cytotoxic responses against tumors (21, 22). Nonsynonymous mutation burden, rather than total mutation burden of exons, was demonstrated to be more closely associated with the clinical advantage of anti-PD-1 treatment, validating the importance of neoantigens in dictating response (23). Tumor mutation burden (TMB) is calculated as the total number of nonsynonymous mutations per DNA Megabase (Mb) (21, 24, 25). Low TMB, or low numbers of clonal neoantigens, presenting reduced tumor immunogenicity, is considered as a primary resistance marker to immunotherapy (15, 26).

Clinically, low TMB or neoantigen load has correlated with inferior response and poor PFS to monotherapy of anti−PD1/PD-L1 antibodies in NSCLC (25, 27–30). However, it fails to predict the clinical outcomes, in regard to overall survival (OS) and combination regimens (31, 32). The influence on the OS by subsequent treatments and the additional complexities to the study of immunotherapy resistance added by combinations may partly explain these controversial findings. Recently, a corrected TMB (cTMB) approach based on the adjustment of tumor purity was developed by Anagnostou and colleagues, which was identified on abundant tumor samples mined from The Cancer Genome Atlas (TCGA) and then confirmed in a patient cohort received ICIs therapy. This cTMB more accurately predicted the outcomes of immunotherapy, suggesting that the TMB in samples with low tumor purity was mistakenly underestimated, which was especially important for metastatic NSCLC, because the tumor purity of tissue samples obtained by bronchoscopy or puncture biopsy was often limited (33).

The dilemma of insufficient tissue sample for TMB assessment in a considerable number of patients with NSCLC has given rise to the employment of peripheral blood TMB (bTMB) as a substitute predictor of response or resistance to ICIs in NSCLC (34). In keeping with what was previously reported in tissue, low bTMB evaluated by different plasma sequencing assays was significantly correlated with poor survival or response to immunotherapy in several retrospective and prospective studies (35–37).

##### Increased neoantigen intratumor heterogeneity

In addition to the TMB or the numbers of clonal neoantigens, increased neoantigen intratumor heterogeneity (ITH, defined as relative fraction of subclonal neoantigens) can also impair the sensitivity to ICIs by elevating the likelihood of selection of subclones with poor immunogenicity (25, 38). The considerable variation of neoantigen heterogeneity was demonstrated by McGranahan and colleagues in seven primary NSCLCs (25). On average, 44% of heterogeneous neoantigens were reported only in a subset of tumor regions. They conducted neoantigen and clonality analysis in lung cancer data from TCGA and then validated the approach in a cohort of NSCLC patients treated with ICIs. Compared with high TMB alone, the combination of high TMB with low ITH seems to have a stronger association with clinical benefit to ICIs in this population.

##### Aberrations in certain oncogene/tumor suppressor genes

Aberrations in oncogenes and tumor suppressor genes can regulate immune response by amending cytokine profile and immune cell composition and thus render tumor cells resistant or sensitive to ICIs.

Generally, alterations in oncogenic driver genes are characterized as resistant markers to immunotherapy. Although epidermal growth factor receptor (EGFR) mutations and anaplastic lymphoma kinase (ALK) rearrangement tend to have high PD-L1 expression due to the activation of signaling pathways (39, 40), the low mutation or neoantigen load (41), along with the following mechanisms, impairs the immunotherapy sensitivity in this group of patients with lung cancer. First, EGFR mutations have the potential to shape an inert immune environment by up-modulating a series of immune suppressors including inhibitory immune checkpoints (e.g., PD-1 and CTLA-4), immunosuppressive cells (macrophages and regulatory T cells), and cytokines (like TGF-β, IL-6, and IL-10) (42, 43). It has been reported that activated EGFR cascade was associated with elevated T-cell exhaustion and reduced cytotoxic T lymphocytes (CTLs) in a lung adenocarcinoma model (40). Second, downstream pathways of EGFR mutation, such as MAPK, PI3K/AKT, and Janus kinase (JAK)/STAT pathway, negatively affect immunoregulation. Other oncogenic driver-genes that frequently have high PD-L1 expression in lung cancer include ROS1 rearrangements (44), MET exon 14 skipping mutations (45), and BRAF mutations (44, 46). In contrast, RET rearrangements (47) and HER2 mutations (44) have been reported recently to exhibit low PD-L1 expression. None of these oncogenotypes demonstrated favorable clinical responses to ICIs monotherapy except for BRAF mutations, either V600E or non-V600E.

STK11 gene inactivation either by mutational or non-mutational machinery is linked to an indolent immune microenvironment with lower Tumor-infiltrating lymphocyte (TILs; CD3+, CD4+, and CD8+ cells) and PD-L1 expression in spite of the existence of moderate to high TMB (48). Inactivated STK11 gene was recently reported to weaken the innate immune responses by epigenetic inhibition of stimulator of IFN genes (STING), suggesting epigenetic silencing is likely to mediate the promotion of T-cell exclusion by the loss of STK11 (49). In line with these findings, it has been observed in several studies that compared with the wild-type gene, STK11 mutation predicted poorer clinical outcomes of immunotherapy in advanced NSLCL (50, 51). The tumor suppressor TP53 mutation, a well-known negative prognostic factor in lung cancer, is found to be associated with increased PD-L1 expression and higher TMB in non-squamous NSCLC (30) and KRAS-mutated lung adenocarcinoma (51).

KRAS-mutated lung cancer presents distinct immune profiles, biology, and therapeutic vulnerabilities in different subsets classified by co-occurring genetic events (50). Generally, KRAS/TP53 co-mutation predicts sensitivity while KRAS/STK11 co-mutation predicts resistance to immunotherapy in NSCLC. Dong et al. identified TP53/KRAS co-mutated subclass exhibited the highest percentage of PD-L1+/CD8A+ and particular increased PD-L1 expression. They further confirmed a remarkable clinical benefit from pembrolizumab in this population (52). Co-mutation of STK11 was shown to cause the accrual of neutrophils with T-cell-suppressive effects, accompanied with an analogous elevation in the production of T-cell depletion biosignatures and tumor-promoting cytokines (50, 53). TIL numbers and the expression of PD-L1 were also decreased (53). Consistent with these preclinical predictions, patients with KRAS/STK11 co-mutation or single mutation of STK11 had poor response and survival compared with those with wild-type when treated with ICIs (51, 54).

The Kelch-like ECG-associated protein 1 (KEAP1) gene, regulating the cellular antioxidant and cytoprotective transcriptional programs, plays a key role in mediating immune evasion in NSCLC. Depletion of KEAP1 is associated with reduced leukocyte infiltration, increased PD-L1 expression and might also influence other immune cells such as NK cell recruitment and function (55, 56). Co-occurring KEAP1 and phosphatase and tensin homolog (PTEN) inactivation represent an immunologically “cold” tumor while concurrent mutations in KEAP1 and STK11 leads to absence of pro-cancerogenic M2 macrophages (57). However, there are conflicting data on the role of KEAP1 mutation and its co-mutation with STK11 in immunotherapy resistance in NSCLC. KEAP1/STK11 co-mutations were verified to correlate with resistance to ICIs in patients with NSCLC despite high TMB (58). Similarly, STK11, and/or KEAP1 genomic variations posited lack of clinical advantages from combination of immunotherapy with chemotherapy in patients with NSCLC (59). However, inconsistent results were reported recently that clinical benefit from pembrolizumab compared to chemotherapy was poorer in the patients with STK11 and KEAP1 mutation compared with those in wild type in Keynote 042 trial, but the response and survival to immunotherapy were not significantly different between mutant and wild subgroups (60).

The WNT/β-catenin pathway is an additional immunotherapy resistance mechanism. A negative relationship was demonstrated between the level of β-catenin and TILs, which was modulated by deficiency in the recruitment of CD103+ dendritic cells (DCs) essential to T-cell priming and reduced expression of the cytokine CCL4, suggesting WNT/β-catenin signaling pathway is likely to mediate ICIs resistance through T-cell exclusion (61).

Similarly, the MAPK/PTEN/PI3K signaling pathway has been identified to be involved in immunotherapy resistance. Loss of PTEN and the bioactivation of the phosphatidylinositol 3-kinase (PI3K) signaling pathway in tumors decrease the activity of CTLs through the recruitment of inhibitory cells to the microenvironment and Vascular endothelial growth factor (VEGF) expression (62, 63), so that they promote resistance to ICIs (63, 64). The association of PTEN deletions or *PIK3CA/AKT* mutations with increased PD-L1 expression and immune resistance was also found in glioma (65). It was shown in preclinical models that a PI3K-γ inhibitor decreased myeloid-derived suppressor cells (MDSCs) and improved response to ICIs (66).

##### DNA repair and replication gene alterations

Genetic instability caused by alterations in DNA replication and repair genes can augment immunogenicity via a high-TMB neoantigen load (67–69). Correspondingly, deficient DNA mismatch repair (dMMR) or high microsatellite instability (H-MSI) are suggested as sensitive predictors to ICI immunotherapy in many tumor types. Beyond high TMB, increased CD8+ TILs were also reported to be associated with alterations in mismatch repair genes (70), *BRCA2* (71), and *POLE* (72) in different tumors. However, the role of these genes in immunoregulation in NSCLC remains to be elucidated.

##### Interferon-gamma signaling mutation

The interferon-gamma (INF-γ) signaling cascade is a crucial component of immunotherapy and tends to serve a critical function in primary, adaptive, and acquired resistance to ICI treatment (73–75). IFN-γ is a critical cytokine secreted by activated T cells, natural killer (NK) T cells, in the cancer microenvironment, and it moderates the immune reaction via the downstream enzymes JAK 1/2 and the signal transducer and activators of transcription (STATs) (76). The INF-γ axis exerts both positive and negative impacts on antitumor immune reactions (77). On one hand, it activates an functional antitumor immune reactive via (1) intensifying antigen presentation by up-modulated secretion of MHC-I; (2) recruiting other immune cells by up-regulation of the expression of chemokines (CXCL9, CXCL10, and CXCL11) with effective chemoattractant impacts on T cells (78); and (3) exerting direct anti-proliferative and pro-apoptotic impacts on cancer cells (79). On the other hand, IFN-γ acts in a negative-feedback axis to elevate PD-L1 expression as well as other crucial immune inhibitory components, including IDO1, down-modulating the cytotoxic reaction and adaptive resistance to cancer cells (80, 81) (Figure 1A).

Additionally, copy-number alterations (CNAs) linked to DNA damage response and regulation of DNA editing/repair gene expression were shown to emanate from the malignant exposure to IFN-γ-secreting antigen-specific CTLs *in vivo*, implying that intensified genetic instability could be among the mechanisms through which CTLs and IFN-γ immunoedits cancers, changing their immune resistance due to genetic evolution (82).

Tumors neutralize the impact of IFN-γ by mutating or down-regulating the molecules involved in the IFN-γ signaling pathway, including IFN-γ receptor chains, regulatory factors, JAK1/2, and STATs upon continuous IFN-γ exposure (73, 83). Multiple studies have demonstrated that mutations of IFN-γ axis and consequent loss of JAK/STAT contribute to immune escape of tumor cells and by that leads to primary or acquired resistance to ICI therapy via incapacity of up-regulating the expression of PD-L1 and MHC-I (73, 78, 84). Any deficiencies in IFN-γ, JAK1/2, or STATs including gene mutations, loss of protein expression, negative regulator presence, or epigenetic silencing would prevent signaling in response to IFN-γ and thereby end up to the up-regulated tumor growth and apoptosis inhibition and down-regulated T-cell infiltration and expression of PD-L1 and MHC-I (74, 78, 85, 86). Correspondingly, genomic changes disturbing IFN-γ pathway genes, including the amplification of suppressor genes PIAS4 and SOCS1 and the deletion of IFNGR1, IFNGR2, IFIT1, IFIT2, IFIT3, IRF1, MTAP, and miR31, have been described as possible machinery of primary resistance to various ICI therapies (73). An IFN-γ-related mRNA profile that contains 10 genes (CCR5, CXCL9, CXCL10, CXCL11, GZMA, HLA-DRA, IDO1, IFNG, PRF1, and STAT1) was additionally identified to predict the response to anti-PD-1 therapy in melanoma (87).

#### Transcriptomic Signatures

In a recent publication, transcriptional signatures, referred to as innate anti-PD-1 resistance (IPRES) with inflammatory and mesenchymal tumor phenotypes, were shown to manifest poor response to anti-PD-1 therapy in metastatic melanoma (88). Approximately 700 differentially expressed genes (DEGs) were identified between the responsive and non-responsive pretreated tumors. Compared with those of responsive tumors, the transcriptomes of non-responsive tumors were dominated by gene up-regulation events. The up-regulated DEGs in non-responsive tumors, considered as T-cell-suppressive, are involved in mesenchymal transition (*TWIST2, TAGLN, FAP, AXL, ROR2, WNT5A*, and *LOXL2*), monocyte/macrophage chemotaxis (*CCL2, CCL7, CCL8*, and *CCL13*), immunosuppression (*IL10, VEGFA*, and *VEGFC*), and angiogenesis and wound healing (89–91). By contrast, down-regulated gene *CDH1* (which is typically down-regulated by mesenchymal tumor cells) was also detected in non-responsive pretreated tumors. Interestingly, there was no difference in the expression of INF-γ pathway signatures, other T-cell-related genes (e.g., *CD8A/B*, *PD-L1*, and *LAG3*), and the genes that presumably modulate immune checkpoint sensitivity between responsive and non-responsive groups, suggesting that T-cell-suppressive inflammatory and mesenchymal phenotypes of tumor are associated with primary resistance to anti-PD-1 therapy.

#### Epigenetic Modification

Emerging evidence has suggested that epigenetic modification may mediate primary resistance and contribute to acquired resistance during ICI therapy through the profound effect on many aspects of antitumor immunity: neoantigen presentation and processing; T-cell functions, differentiation, and proliferation; memory T-cell phenotype acquisition; interfering with T-cell migration; and mediating T-cell exhaustion (10, 92–94).

Epigenetic targeting agents, including those targeting histone deacetylation or methylation as well as targeting DNA methylation, have exhibited encouraging antitumor activity either as monotherapy or in combination with immunotherapy in preclinical studies (94, 95). Clinical trials investigating the performance of these agents combined with adaptive T-cell transfer (ACT) in patients with acquired resistance to prior immunotherapy are ongoing (96).

#### Stability of Chromatin Remodeling Complexes

Stability of chromatin remodeling complexes within tumor cells can also contribute to immunotherapy resistance by multiple mechanisms. It was found that tumor cells were more sensitive to CTL killing, which leads to increased response to anti-PD-1/PD-L1 therapy, due to the deficiency in chromatin remodeling complex SWI/SNF (97, 98). BRG1-associated factor (BAF) and polybromo-associated BAF (PBAF), as the mammalian analogs of the SWI/SNF complex, are essential tumor suppressors and loss of function (LOF) mutations of them were shown to sensitize tumor cells to ICI therapy (98). The inactivated PBAF subunits exhibited elevated CXCL9/CXCL10 expression and TILs recruitment as a result of increase of chromatin accessibility to transcriptional regulators of IFN-γ-inducible genes (97). ARID1A/B subunits are unique to BAF, while other subunits (ARID2, BRD7, and PBRM1) are exclusively contained by PBAF, despite the high similarity of these complexes (99). In another study, loss of ARID1A was found to elevate MSI by defective recruitment of mismatch repair genes and thus increase TMB, which eventual sensitize tumor cells to PD-L1 inhibitor (100).

#### Absent Tumor PD-L1 Expression

The PD-1/PD-L1 axis represents one of the foremost mechanisms of modulation of peripheral immune tolerance as well as T-cell activation. Up-regulation of PD-L1 by cancer cells and antigen-presenting cells (APCs) is one approach through which tumors avoid immunosurveillance and constitutes the principle behind PD-1/PD-L1 blockade therapies (101). Absent PD-L expression of tumors has been found to be generally associated with less responses and inferior survival benefits to anti-PD-1/PD-L1 therapies compared with higher expression (102) and may serve as a resistant marker. However, up to 20% of PD−L1−negative malignancies showed responses to PD−1 inhibitors in some cohorts (103), as PD-L1 expression can be up-regulated by other factors including activated IFN-γ cascade (will be discussed in a separated part), suggesting tumor PD-L1 expression alone is not dependable at predicting outcomes of PD-1/PD-L1 inhibitors.

Any factors that affect the PD-L1 expression of tumor cells may lead to resistance to immunotherapy. Beyond encoding genes, PD-L1 expression can be affected by the mutational features of tumor although it is not paralleled with TMB in most of the tumors (104–106). The inherent mechanisms, which have been shown to result in constitutive expression of PD-L1 by tumor cells, consist of alterations in the PTEN/PI3K/AKT pathway (65, 107), MYC overexpression (108), EGFR mutations (40), CDK5 truncation (109), and elevated PD-L1 transcripts stabilized by disruption of the 3-untranslated region (UTR) of this gene (110). Tumor-specific immune response may also be affected by constitutive expression of PD-L1 caused by these oncogenic signaling processes on tumor cell surface. Although it is still unclear whether it causes an increased or decreased possibility of responding to anti-PD-1/PD-L1 therapies, the constitutive PD-L1 expression could result into inadequate response to other immunotherapeutic approaches by suppressing antitumor effect of T cells. The other transcriptional factors constituting HIF1, NFkB, and STAT3, as well as epigenetic factors, additionally participate in the modulation of PD-L1 expression (111).

Inflammatory and hypoxic tumor microenvironment (TME) can also lead to PD-L1 expression on many cell types including tumor cells by Toll-like receptor (TLR) ligands. The recruitment of activated T cells can increase the inflammatory mediators and successively induce the PD-L1 expression on the surface of tumor cells. These tumor cells specifically locate at the invasive periphery where T cells are often abundant (112, 113).

Besides, PD-L1 is stabilized through N-glycosylation and palmitoylation (114, 115). This is crucial for its interaction with PD-1. The resistance to anti-PD-1/PD-L1 treatment could moreover be attributed to the degree of generation and secretion of soluble forms of PD-L1. These variants without the transmembrane domain because of alternative splicing have been reported in recurrent NSCLC incidences that re-occurred after anti-PD-L1 antibody therapy with the ability to act as soluble imitates for anti-PD-L1 antibodies (116).

#### Deficiency in Antigen Presentation

##### Loss of neoantigen

Loss of neoantigens in the context of immune-mediated pressure is postulated to be another mechanism leading to resistance. In the concept of immunoediting, the constant interactions between tumor cells and the immune system trigger the production of subclones that do not express neoantigens, consequently conferring poor immunogenicity and resistance to ICIs (117). It was demonstrated by Anagnostou and colleagues that seven to eight putative neoantigens were lost in the recurrent NSCLC after ICI treatment, suggesting that immunoediting plays a role in acquired resistance to immunotherapy (118). T-cell-mediated neoantigen immunoediting can be induced by the dynamic interactions between T cells and tumor cells, consequently causing partial or total loss of neoantigen (119). Consistently, deficiency in genes that encode target tumor antigens was demonstrated to be associated with acquired resistance in a murine model treated with adoptive T-cell therapy (ACT) in melanoma (120). However, this relationship between acquired resistance and loss of target neoantigens was not observed in a single patient case who achieved a complete response to ACT in a separate study (121), suggesting that down-regulation/loss of neoantigens may occur during immunotherapy, but should be taken as a canonical mechanism of acquired immune resistance.

Proinflammatory cytokines are likely to contribute to immune escape by inducing loss of antigen expression, resulting in acquired resistance too. The process of Tumor necrosis factor-α (TNF-α)-induced epithelial-to-mesenchymal de-differentiation was shown to lead to a loss of neoantigens causing transformation to a tumor phenotype that is less immunogenic and can more readily evade immune surveillance in the ACT-treated mouse model in melanoma (122). Other TIL generated cytokines, such as IL-6 or TGF-β, are also shown to be involved in the induction of epithelial-to-mesenchymal transition in mouse models across numerous types of tumors, indicating that acquired resistance can be promoted by inflammation.

##### Defective neoantigen presentation

Defective neoantigen presentation serves a crucial function in ICI acquired resistance. The alterations in this process could happen in beta-2-microglobulin (B2M), transporters associated with antigen processing (TAP), or MHC itself (123, 124) (Figure 1A).

As part of the MHC class I (MHC-I), B2M is crucial during antigen presentation and its genetic deficiency, including loss of heterozygosity (LOH) and deletions or point mutations, was identified to be an important route for primary and acquired resistance to ICIs (125, 126). Other defects that would affect neoantigen presentation include T-cell receptor (TCR) binding domain mutations of MHC-I (127), loss of tapasin (a MHC-I antigen processing molecule), selective epigenetic silencing of the human leukocyte antigen (HLA) A3 antigen, loss of one HLA haplotype (128, 129), and LOH in HLA (130). Homozygosity in one or more of the three highly variable genes (HLA-A, HLA-B, and HLA-C) that encode MHC-I, which are likely to restrict neoantigen presentation to CTLs, was identified to have a significant association between resistance to ICI therapy in a large cohort of cancer patients (131). In contrast to anti-CTLA-4, the expression of MHC-II (but not MHC-I) proteins by tumor and the presence of IFN-γ-mediated gene signatures were found to be associated with the positive responses to anti-PD-1 therapy in melanoma (132).

Defective neoantigen presentation may be mediated by IFN-γ signaling pathway through JAK1/2 and the STATs, by down-regulating the expression of MHC-I (133). Actually, the IFN-γ pathway has both unfavorable and favorable impacts on antitumor immune responses and plays a key role in acquired and primary resistance to ICI therapy (as discussed above).

### Tumor Cell-Extrinsic Immune Landscape

#### T-Cell-Related Factors Involved in Tumor-Cancer Immune Cycle

##### Tumor-infiltrating lymphocytes

Tumor-infiltrating lymphocytes constitute a complex group of immune cells with distinct functions and different clinical impacts. Among them, tumor-specific CD8+ T cells can execute anti-cancer activities by killing tumor cells directly and has a strong prognostic effect in NSCLC (134, 135). CD4+ cells are composed of a group of lymphocytes (Tregs CD4+, Th1, Th2, and Th17) secreting diverse cytokine to activate and suppress CD8+ cells. Th1 secretes IFN-γ and IL2, while Th2 secretes IL-4, IL-5, IL-9, IL-10, IL-13, and IL-25 (136, 137). CD45RO+ T cells, also known as memory T lymphocytes, are another subclass of TILs. Regulator and memory T lymphocytes will be discussed in *section* “Suppressive tumor microenvironment.”

Low CD8+ TIL density was correlated with impaired efficacy and survival in NSCLC patients treated with ICIs (138), suggesting that immunotherapy resistance was mediated by low TILs but was then positively modulated by PD-L1. TILs can be assessed by immunohistochemistry or standard hematoxylin and eosin (H&E) staining; however, no consensus has been reached hitherto in the various scoring models using H&E staining in NSCLC (139–142). A radiomic fingerprint of CD8+ TIL derived via computerized tomography was developed recently and showed promising efficacy in predicting response to ICI therapies but requires further validation (143).

Thus, tumors can be described as three main immune organization profiles (hot, altered, and cold) as per the presence of TILs and correlated proinflammatory cytokines (144). The “cold” immune tumor is characterized as absence of TIL within and at the edges of tumor, manifesting resistance to immunotherapy either due to absent immune stimulation (as with low neoantigen cancer’s poor antigen presentation) or because of failed T-cell priming (as with intrinsic insensitivity to T-cell killing). The “altered” immune tumor is characterized as low TIL within the tumor (“immunosuppressed”) or high TIL at the edges of the tumor (“excluded”), whereas “hot” is high degree of TIL (144). Recently, intratumorally geospatial heterogeneity of TIL was revealed in NSCLC. Tumor subclones from “cold” immune regions were related to mutation space more closely and diversifying more recently compared with those from “hot” immune regions. Higher risk of recurrence was observed in tumors with more than one “cold” immune region (145).

##### Impaired T-cell priming and infiltration

Reduced proliferation and inadequate diversification of T cells possibly contribute to ICI resistance. Impeded priming of naive T cells by blocked DCs recruitment was demonstrated in melanoma to be correlated to the lack of TILs and ICIs resistance (146). The function of DCs can be potentially influenced by the cytokines in the TME through (1) impaired migratory capacity as well as decreased synthesis of costimulatory components (CD86/80) by TGF-β (147, 148); (2) prevented DCs maturation by IL-6-gp130-STAT3 axis; and (3) inhibited activity by Indoleamine 2,3-dioxygenase 1 (IDO, will be discussed in *Section* “Suppressive tumor microenvironment”). IFN-α signaling pathway is important to the priming of T cells by DCs. It was found that TME with remarkable insufficient IFN-α-producing DCs naturally led to lessened antitumor T-cell priming and thus resistance to ICIs (149, 150). Activated IFN-α stimulated production of the chemokine CXCL10 to recruit TILs to tumor beds and in turn initiate spontaneous antitumor T-cell response (149–151). Preliminary trials combining IFN-α 2b therapy with anti-CTLA-4 inhibitors have indicated clinical activity, which could be caused by diminished populations of MDSC (152, 153). Combinations of other ICIs and IFN-α 2b are currently investigated (154).

Immune resistance also occurs if the tumors evolve the ability to prevent infiltration even if tumor-specific Teffs are formed. Mechanisms that lead to impaired T-cell infiltration involve components in the epigenetic silencing of immune cells (155) and the modification of secreted chemokines (156, 157). Transcriptional program that is associated with T-cell exclusion and thereby predictive resistance to anti-PD-1 therapy was identified in melanoma (158). Stromal cells surrounding tumors within TME can develop the capacity to obstruct effector T-cell entry, and the TGF-β cascade appears to serve a crucial role in promoting T-cell exclusion features in peritumoral fibroblasts (123, 159).

##### T-cell receptor clonality

T-cell receptor clonality is emerging as a new biomarker to predict the resistance and immune-related adverse events to ICIs therapy. Since baseline CD8+ T-cell density was found to overlap between respondents and non-respondents to ICI therapy (160–162), it led to the speculation that a constrict TCR arsenal possessed by the baseline T cells concentrated on the antitumor immune reaction and is associated with response to ICI therapies. T-cell clones can be identified by detecting TCR rearrangements constituting genes in the variable (V)-diversity (D)-joining (J) region, which generate the antigen-specific complementarity-determining region 3 (CDR3). The responsivity of TCRs generated by TILs determines their potential to interplay with tumor antigens that are presented on APCs. Thus, the assessment of T-cell clonality divulges the extent of T-cell expansions caused by tumor antigens and contributes to explore the mechanisms underlying T-cell toleration to tumor antigens.

A lower baseline clonal T-cell arsenal has been shown to be linked to worse clinical benefits to ICIs and survival in cancer patients (162, 163). Besides, a remarkable increase in T-cell clones was reported in responders during anti-PD-1 therapy compared to non-responders, implying a cancer-specific reaction to immunotherapy for these patients. Moreover, baseline TCR clonality did not strongly associate with TIL density, implying that low-TIL density tumors could still respond to anti-PD-1 treatment if TIL has a narrow TCR clonality specific to the tumor antigen (164). Inconsistently, it was recently found that T-cell clonality had a positive relationship with T-cell density, PD-L1 expression, and TMB, and a negative relationship with EGFR mutation in NSCLC (165). A corresponding relationship was found between the number of TCR sequences and the number of nonsynonymous mutations, spatial heterogeneity in expanded TCR repertoire, and spatial mutational heterogeneity within tumors in NSCLC, respectively. This intratumorally spatial heterogeneity of TCR repertoire maps the neoantigen landscape, sculptured by focal antigen processing defects or HLA loss (166). Thereby, further investigations to identify the role of TCR clonality in immunotherapy are required.

##### Alternate immune checkpoint receptor up-regulation

Compensatory up-regulation of numerous alternate immune checkpoint receptors during ICI therapy as a result of the activation of diverse cellular signals and IFN-γ signaling pathway were observed across multiple studies and have been characterized to be linked to ICI adaptive resistance in NSCLC (84, 167, 168). The expression of CD8+ T cells harboring receptors showed serious flaws in proliferation, migration, and cytokine secretion, indicating their immunosuppressive capacity. In addition, progressive T-cell exhaustion was found in the tumors with highly expressive or co-expressive receptors and different receptor displayed different exhausted phenotype (167).

Among these receptors, lymphocyte activation gene 3 (LAG-3) has great potentiality in cancer immunotherapy because co-expression of PD-1 and LAG-3 was often found in T-cell-depleted immune microenvironment, and PD-1 inhibitors combined with LAG-3 blockades showed strong synergic antitumor responses in preliminary models (169). LAG-3 is a co-inhibitory receptor extensively expressed in TILs in various tumors and serves a crucial function in mediating immune escape by suppressing T-cell antitumor functions. It exerts immunosuppression via binding to MHC-II molecules and other ligands such as galectin-3 and fibrinogen-like protein 1 (FGL1) (170). Thus, blocking LAG-3 can restore antitumor immunity and the combined LAG-3 inhibitors therapy may accordingly overcome immunotherapy resistance. In addition, the expression of these ligands, on the basis of the receptor–ligand interactions, may serve as important biomarkers to predict the efficacy of LAG-3 blockades in lung cancer (167).

The other alternative immune checkpoint receptors, e.g., T-cell immunoglobulin and mucin-3 (TIM-3), V-domain immunoglobulin-containing suppressor of T-cell activation (VISTA), B and T lymphocyte attenuator (BTLA; also referred to as CD272), T-cell immunoreceptor tyrosine-based inhibition motif domain (TIGIT), and sialic acid-binding Ig-like lectin 9 (SIGLEC9), have been discovered (144). Thus, these alternate immune checkpoints are likely to be combined with existing ICI therapy to conquer the resistance. Increased efficacy of PD-1 inhibitors combined with anti-TIM-3 or anti-LAG-3 regimens has been observed in either pre-clinical models or phase I clinical trials (171, 172). Currently, numerous clinical trials evaluating the therapeutic impact of alternate immune checkpoint blockade applied on its own or in combination with PD-1/PD-L1 inhibitors in multiple malignances are ongoing.

##### T-cell exhaustion and phenotype alteration

T-cell exhaustion is another factor involved in the primary and acquired resistance to ICI therapy (Figure 1C). Exhausted T cells exhibit impaired activity with progressive LOF and antigen persistence compared with Teffs and can be induced by the PD-1/PD-L1 interactions (173). Chronic exposure to cognate antigen triggers increased expression of PD-1, which results in the accumulation of T-cell exhaustion and thus T-cell dysfunction (174). The presence of PD-1 high expression can either exist prior to PD-1 inhibitors, which is associated with primary resistance partially depending on tumor-associated regulatory T cells (Tregs), or develop after the anti-PD-1 therapy, which leads to acquired resistance by severe T-cell exhaustion. In contrast, studies showed that the exhausted T cells with PD-1 low to intermediate phenotype retain the capacity to be reinvigorated by ICIs (158, 175). Epigenetic alterations were found to be associated with T-cell exhaustion too recently. Exhausted T cell displayed a unique chromatin landscape, which alters the transcriptional state, limits its effect function, and determines its capacity to be reprogrammed after therapeutic intervention (176–178).

The formation of memory T cells is crucial to the avoidance of tumor relapse and therapy resistance following drug withdrawal, especially in the long-lasting duration of responses to ICI therapy. Research evidence shows that patients with resistance to anti-PD-1 treatment have fewer tumor-correlated memory T cells relative to sensitive patients (179). Memory T cells remain dormant until antigen re-challenge (180, 181) and if precursor memory T cells are exhausted under chronic antigen exposure, it will lead to memory T-cell deletion and lack of formation (173, 177).

Acquired resistance can be mediated by the alteration from cytotoxic activity to inactivity phenotype of antitumor T cells during TCR-engineered ACT. The original highly cytolytic profile when administrated to patients, which showed strong efficacy initially, was reported to change to a phenotype with impaired cytotoxic functions and Th2-related cytokine release when tumor relapses within months (182, 183).

#### Suppressive Tumor Microenvironment

##### Increased immunosuppressive cells

The TME is a complex net consisting of a variety of immune and stromal cells, cytokines, extracellular matrix, and vasculature, which affect response to immunotherapy. Immune-suppressive cells, including Tregs, MDSCs, M2 macrophages, along with inhibitory cytokines in the TME, can contribute to the inhibition to immune responses (136, 184) (Figure 1C).

Tregs can inhibit Teff reactions by secreting certain inhibitory cytokines (IL-10, IL-35, and TGF-β) or by direct cell contact (185–187). The cytokine IL-10 influences antigen presentation by down-regulating the expression of MHC-II and co-stimulatory components on DCs, thus intercepting the Teff activation (187). The ratio of Teffs to Tregs was shown to be related to the responses to ICIs in mouse models, in that incapacity of either increasing Teffs or decreasing Tregs may cause resistance to immunotherapy (188, 189). Factors that affect Tregs activity, at the same time, are putative biomarkers of resistance. For instance, soluble CD25, an IL-2 receptor whose binding is assumed to stimulate Treg proliferation, was established as a negative predictor of OS for patients treated with anti-CTLA−4 (190). However, tumor-infiltrating Tregs might likely coexist with multiple immune cells, insinuating a potential immunoreactivity. It was reported that a high baseline expression of FoxP3+ Tregs in the tumor is positively associated with better survival in a retrospective study involving patients under the treatment of anti-CTLA-4 antibodies (161).

Myeloid-derived suppressor cells promote immune evasion and tumor growth and have emerged as critical modulators of immune responses in cancer. Studies have suggested the existence of MDSCs in TME correlates with reduced efficacy of immunotherapies, including ICIs therapy (191), ACT (192), and DC vaccination (193). Therefore, reprogramming or eradicating MDSCs might improve clinical response to immunotherapy.

Tumor-associated macrophages (TAMs) can be classified into M1 and M2 macrophages according to disparities in surface molecules, expression of transcription factors, cytokine profiles, and metabolism (194, 195). They promote antitumor immunity effects (mediated by M1) and pro-tumorigenic properties (mediated by M2) that modify the TME (196). The role of TAMs in mediating immunotherapeutic resistance in tumor has been discussed in several reports (197, 198). It was indicated to directly inhibit T-cell responses through PD-L1 in preclinical studies of liver (199) and ovarian cancer (200). The inhibitor of CSF-1R, a receptor for macrophage colony-stimulating growth factor, was investigated in mouse models of pancreatic cancer where it decreased the frequencies of TAMs, while increasing IFN production and delaying tumor progression (201, 202). Similarly, CSF-1R inhibitor was found to synergize ACT therapy in a melanoma model (203). These data suggest that CSF-1R inhibitor may overcome the resistance to immunotherapy.

Specific chemokines, such as CCL5, CCL7, and CXCL8, play an important role in the recruitment of Tregs and MDSCs to the TME, consequently boosting an immunosuppressive climate (204). Alternately, chemokines CXCL9 and CXCL10 recruit CTLs to the TME (205) and the epigenetic silencing of the genes encoding them can reduce TILs and consequently promote resistance to ICIs (205). Epigenetic modulators of these chemokine receptors relieved the suppression of these Th1-cell-type chemokines and increased TILs leading to improved therapeutic efficacy of PD-L1 inhibitor in a model for ovarian cancer (155).

##### Elevated immunosuppressive cytokines

The cytokine milieu is critical to the recruitment, activation, and proliferation of immune cell, performing both immune stimulatory and suppressive effects (206). Transforming growth factor-β (TGF-β) is a cytokine playing key roles in angiogenesis and immunosuppression by stimulating Tregs (207) and excluding T cell in peritumoral fibroblasts (123, 159). Up-regulated TGF-β signaling was correlated with poorly immunogenic tumors and restrained response to ICIs in a colorectal cancer model, indicating resistance to therapy (159). Consequently, enhanced antitumor response to ICIs was observed following application of TGF-β inhibitor either alone or in combination with anti-CTLA-4 or radiation therapy (208, 209). Bintrafusp alfa, a bifunctional fusion protein composed of the extracellular domain of TGF-β receptor II (a TGF-β “trap”) fused to a human immunoglobulin G1 antibody blocking PD-L1, demonstrated favorable efficacy in patients with advanced NSCLC. Ongoing phase III trial is expected to validate the efficacy of bintrafusp alfa vs. pembrolizumab in the first-line setting in advanced NSCLC (NCT03631706).

Tumor necrosis factor-α pathway is postulated to be another immune evasion machinery conferring resistance to PD1 blockade. The expression of TNFα in an inflamed TME positively correlates with the expression of PD-L1 and TIM-3, along with impaired accumulation and increased activation-induced death of CD8+ TILs in melanoma models treated with anti-PD1 therapy. Accordingly, inhibition of TNF-α prevents the expression of PD-L1 and TIM3 and hampers anti-PD1-induced TIL death (210). Therefore, this study offers a rationale for the combination of PD-1/PD-L1 inhibitors with TNFα blockade as a novel immunotherapeutic strategy to overcome resistance in lung cancer, and the phase I clinical trial testing the combination is ongoing (NCT03293784).

Vascular endothelial growth factor has been linked to both decreased T−cell infiltration and immunosuppressive effects in addition to promoting angiogenesis and thus is associated with resistance to ICIs (211). Multiple mechanisms are involved in the interaction of VEGF with antitumor immunity: (1) VEGF prevented the commitment of lymphoid progenitors, decreasing progression to the T-cell lineage (212); (2) VEGF signaling promotes the infiltration of Tregs through a selective endothelium and reduces trafficking and extravasation of CTLs into the TME (213); and (3) VEGF increases expression of inhibitory receptors, contributing to CTL exhaustion (214). Higher levels of VEGF were found in anti-PD-1-resistant patients than sensitive ones (160). Based on these findings and the synergy between angiogenesis blockade and ICI therapies observed in preliminary studies, multiple trials of combination therapy are underway, including bevacizumab and VEGFR-TKI with anti-PD-1 therapy.

Higher levels of interleukin 6 (IL-6) and interleukin 8 (IL-8) have been found recently to be linked to reduced responses and worse clinical outcomes to ICI therapies across multiple types of cancers (215–217). IL-6 is a proinflammatory cytokine generated by T cells and macrophages and is usually involved in the immunoregulation connected to the IFN-γ signaling pathway. It can reduce the expression of PD-L1 and MHC-I, and result in tumor escape and resistance to ICI therapy (218). IL-8 is a proinflammatory chemokine and a chemoattractant for myeloid leukocytes expressed in multiple cancers (219, 220). It potently regulates the chemotaxis of neutrophils (221, 222) and exerts direct pro-tumorigenic effects (223). High levels of IL-8 are regarded to be associated with more neutrophil and monocyte infiltration, defective T-cell functions, and impaired antigen presentation, which subsequently result in resistance to ICI therapy (216, 217).

##### Additional immunoregulative molecules

Contributions from inflammatory processes could participate in quashing the desired impacts of ICIs (Figure 1C). Adenosine can be produced under the condition of hypoxia and ischemia caused by tumor inflammation. It was reported to inhibit the cytotoxic function and proliferation of T cells via the A2A receptor on T cells (224). CD73, which mediates the generation of adenosine through dephosphorylation of adenosine monophosphate, was also demonstrated to suppress immune function (225). CD73 overexpression promotes T-cell exhaustion and is linked to the resistance to ICIs (226, 227).

IDO1 expressed in myeloid cells and cancer cells is a rate-limiting enzyme that converts tryptophan to its immunosuppressive metabolite kynurenine. This enzyme can induce T-cell anergy and apoptosis by gathering kynurenines and consuming the essential amino acid tryptophan and prevent the T-cell clonal expansion (228). It is particularly activated in DCs after binding with CTLA−4 and can be unregulated by CTLA−4 during adaptive immune resistance (229). Reduced expression of IDO at baseline was noted to be associated with poor response to ipilimumab in a phase II study in melanoma (161). Low level of IDO is likely to manifest insufficiency of suppressed TILs feasible to be reactivated by immunotherapy. Correspondingly, IDO-knockout mice exhibited improved OS with ICI compared with wild-type mice, and ICI therapy combined with IDO inhibitors showed both increased numbers and functions of TILs in the TME in an experimental setting (230, 231). However, despite encouraging results observed in preclinical and early-phase clinical studies in different types of tumors, no difference was shown in pembrolizumab combined with IDO1-selective inhibitor vs with placebo in a phase III study in metastatic melanoma (232).

B7-H4 has been proposed as another resistance marker to ICIs recently due to its negative modulation of T cells. B7-H4 constitutes a type I transmembrane protein of the B7 immunoglobulin superfamily and is encoded by the V-set domain containing T-cell activation inhibitor 1 (VTCN 1) gene. It is induced by activated T lymphocytes and down-regulates T-cell function by inhibiting proliferation, cytotoxicity activity, and interleukin secretion, after binding with T cells (233–235). Positive B7-H4 protein expression in patients with advanced NSCLC treated with nivolumab was recently reported to have an enhanced risk of tumor progression and tumor-related death compared with negative expression (236).

### Host-Related Characteristics

Host-related characteristics, including gut microbiome, diet, and antibiotic exposure that adversely affect the gut microbiome, diet, steroid use, vaccine exposure, inflammation state, and autoimmunity, have been shown to relate to primary and acquired resistance to ICI therapy in lung cancer (237, 238) (Figure 1B).

#### Gut Microbiome

Evidence is arising to support the vigorous impact of the gut microbiome on immunotherapy resistance. Multiple studies have demonstrated that less bacterial diversity and lack of enrichment of specific species showed a significant correlation with resistance to ICI therapies. Relative abundance of *Bacteroidales* was found in non-responders, while responders were more likely to have *Faecalibacterium* and *Ruminococcaceae* (239, 240). Furthermore, transplanting the responder’s feces into aseptic mice exhibited improvement in the treatment of PD-L1 inhibitor (241). Certain species can be altered by the antibiotic’s exposure, which partly explained modified response to ICI therapy either in a good or in a bad way (242–244).

The alteration tendency of gut microbiome structure or dominant bacteria may differentially affect the T-cell immune response. It may be due to the cross-reactivity between intestinal microbiota and tumor-associated antigens, enhancing the inflammatory cytokine production, activation of DCs, and antigen presentation (245, 246). IFN-γ-producing CD8+ T cells were successfully induced from a consortium of 11 bacteria in the intestine, and colonization with this 11-bacterial mixture resulted in enhanced efficacy of ICIs in mouse models (247). Consistently, “good” bacteria introduction was reported to significantly increase IFN-γ production in spleen and tumor-draining lymph nodes (TDLN) (246) and induce DCs to secrete IL-12, resulting in increased recruitment of CCR9+CXCR3+CD4+ T cells into tumor beds (244). The activation of DCs was reported to be modulated by the gut microbiome in animal models and cancer patients. The resistance to anti-CTLA-4 therapy can be reversed by oral administration of *Bacteroides fragilis*, which induced Th1 immune response in TDLN and promoted DCs maturation (243). *Bifidobacterium*-feeding mouse presented higher expression of MHC-II in DCs within tumors (248). It remains controversial whether intestinal microbes lead to immunotherapy resistance by affecting the production of Tregs. A higher level of peripheral Tregs was found in patients with “bad” bacteria and was associated with poor response to ipilimumab in metastatic melanoma patients (249). *B. fragilis* can produce a microbial molecule, polysaccharide A, which can promote the formation of the inducible population of CD4+Foxp3+Tregs (a subset of Tregs), thereby negatively regulating the immune system (250), whereas the other two studies reported no differences in Treg differentiation between pancreatic duct adenosarcoma-bearing mice and control, and the number of Foxp3+ T cells between *Bifidobacterium*- and PBS-feeding mice (251, 252). The majority of chemokine genes were reported to be up-regulated by specific species of gut microbiome including *Fusobacterium nucleatum*, *B. fragilis*, and *Escherichia coli* in colorectal cancer cells. Additionally, the gut microbiota-derived microbial load was associated with increased chemokine production (251).

The gut microbiome also acts as an instructive modulator of mutant TP53, which ultimately affects tumor proliferation and the immune system. Kadoshi et al. recently found that mutant TP53 presented contrasting effects in different segments of the gut in a mouse model: a remarkable tumor-suppressive effect in proximal gut and the expected oncogenic effect in distal gut (253). The gut microbiome and its single metabolite gallic acid turned mutant TP53 from a tumor-suppressive effect to an oncogenic one, suggesting that the function of mutant TP53 is plastic and under the control of microbiome and microbiota-derived metabolites.

#### Concomitant Medications

Antibiotics exposure has been reported to be associated with inferior clinical outcomes during ICIs therapy in NSCLC (244, 254). However, it remains debated if antibiotics exposure represents an independent predictive biomarker of ICIs therapy or it is a surrogate for patients with worse prognosis (e.g., poorer performance status, higher comorbidities). The use of antibiotics has an unfavorable impact on the recolonization and subsequent alterations in microbiota composition, which eventually leads to a decline in microbial symbiotic diversity (255). The antibiotics-induced dysbiosis destroys the gut homeostasis and extends from childhood to adulthood, with long-lasting adverse effects on the immune system as well as body metabolism (256–258). The antitumor immune response-induced cyclophosphamide was impaired by antibiotics exposure in the fibrosarcoma model, which was associated with an improvement in the Teff-to-Treg ratio and the loss of *Enterococcus hirae*-elicited helper T cell in tumor immune infiltrate (259, 260). Correspondingly, the absolute numbers of neutrophils and monocytes decreased after oral administration of imipenem, vancomycin, and neomycin before tumor inoculation in lymphoma and colon models treated with oxaliplatin or IL-10 inhibitor (261). On the contrary, the growth of *Fusobacterium* spp.-containing tumor was slowed down after the administration of metronidazole in the mouse model of colon cancer, suggesting such bacteria promote tumor progression (262).

Long-term use of steroids adversely impacts the efficacy of ICIs because of their supposed anti-inflammatory and immunosuppressive effects that potentially hamper the mode of ICI action (263), whereas a transient use of steroids aimed at management of ir-AEs did not negatively affect patients’ survival outcomes (264, 265). Beyond affecting gut microbiome, steroids are known to prevent the activation of T lymphocytes, inhibit the amplification of T helper subsets, recruit Tregs, and promote M2 macrophage polarization (266, 267). Hence, early use of steroids on ICI treatment may prevent this phase of T-cell recruitment and thus impair effective antitumor immune response. The increment of neutrophil-to-lymphocyte ratio (NLR), derived NLR (dNLR), and absolute neutrophil count (ANC) after steroids has been shown to be associated with unfavorable clinical outcome in NSCLC patients treated with ICIs (268). Steroids-induced imbalance of immune cells in TME, especially increased MDSCs resulting in elevated ANC and NLR, is the intermediate of immunotherapy resistance in NSCLC (269).

#### Diet

Diet can affect tumor growth within TME through systemic or local effects in multiple ways. First, it may alter the composition and diversity of gut microbiome, which in turn exerts drastically different effects on host immune function. Second, specific ingredients (e.g., vitamins) may be regulated by dietary patterns and then have an impact on immune status. It is well-known that the general metabolic status determining deviations from ideal body weight, as well as the metabolic factors (e.g., low-level arginine and tryptophan, high-level lactate, and the adenosine pathway induced by increased glucose metabolism), highly influences the immune activity (270–272). An appropriate diet can maintain the homeostatic equilibrium between the inflammatory cascade triggered by Th17 cells and the anti-inflammatory pathway mainly based on the activity of Treg (273).

#### Additional Factors

Chronic accumulation and production of inflammatory molecules in the chronic inflammatory status lead to an immunosuppressant state, which is linked to immunotherapy resistance. Proinflammatory and carcinogenic mediators such as IL-6, TNF-α, and chemokines are released in the TME and tend to trigger a variety of molecular signaling cascades including PI3K/MAPK, JAK/STAT, and WNT/B-catenin, which are involved in the resistance to immunotherapy as mentioned previously. The components of immune cells are also altered to be more immunosuppressive with more TAMs, Tregs, and tumor-associated neutrophils within TME (274). Therefore, blocking inflammation might be an effective strategy to improve the outcome of immunotherapy in NSCLC (275).

Tumor development and autoimmunity are two opposite results of imbalanced immune homeostasis in controlling tumor cell growth (low immune responses) and regulating autoreactive responses (immune overreaction). The host autoimmunity affects the efficacy of immunotherapy in bringing more ir-AEs when too strong or incapacity to prime and activate immune cells when too weak (276). In addition, autoimmunity has an inextricable link with host gut microbiome and anti-microbial immunity, as effector responses that lead to inflammatory tissue damage are the same as those that mediate effective host defense (277).

The relationship between smoking and the efficacy of ICIs remains controversial (278–281). Smoking is associated with high TMB, especially nonsynonymous mutations, subsequently enhances the immunogenicity of tumor, and improves the outcome of ICIs therapy (23, 282). Additionally, PD-L1 expression can be up-regulated by smoking through oxidative stress-dependent mechanism (283) and induced by cigarette smoke and the carcinogen benzopyrene (BaP) via aryl hydrocarbon receptor (AhR) (284). Moreover, smoking may also have an impact on the status of TILs (285) and other immune modulators such as B7-H3 (CD276) (286) and in turn affect the efficacy of ICIs therapy.

## Therapeutic Approaches to Conquer Immunotherapy Resistance

Research and design of therapies to conquer immunotherapy resistance has been advancing along with mechanistic investigations. Combinatory treatments, either via combinations of diverse immunotherapeutic agents or through combinations with traditional treatments, developed to revitalize the defense system with complementing/synergetic mechanisms, have been introduced to serve as alternative approaches for NSCLC therapy. Diverse targets discussed herein have the potential to serve as both biomarkers of resistance and combination therapy targets. In view of the different resistance mechanisms, the combinatory therapy strategies are mainly manifested in the following aspects (the examples of ongoing studies trying to reverse resistance are summarized in Supplementary Table 1):

### Enhance Tumor Immunogenicity

1. Emerging evidence has indicated the positive immunologic effects of chemotherapy (287). On one hand, it regulates the composition and function of immune cells such as CTLs (288), MDSCs, and Tregs (289) in the TME and the molecules expressed on tumor cells; on the other hand, it restores the recognition of immune system to tumor through enhancing tumor antigen presentation via up-regulating the expression of MHC-I and through boosting antitumor immune responses via chemo-induced tumor cell apoptosis (290–292). Multiple randomized phase III clinical trials have compared the combination of chemotherapy and ICIs with chemotherapy alone in treatment-naïve advanced lung cancer (4, 9, 293). The results consistently showed that the combination strategies are superior to chemotherapy alone in the first-line setting, regardless of PD-L1 expression, suggesting that the synergistic activity between chemotherapy and ICIs may offset the insensitivity due to low PD-L1 expression.
2. Similar to the effects induced by chemotherapy, radiation therapy combined with ICIs leads to long-lasting tumor regression through escalating antigen exposure secondary to cancer cell apoptosis, enhancing an inflamed TME (294), raising DCs activation and up-regulating proinflammatory cytokines, causing elevated TILs (295), and facilitating cancer relapsing by non-redundant immune mechanisms (296, 297). Consolidative PD-L1 inhibition after concurrent chemo-radiation significantly improved survival in unresectable stage III NSCLC in the PACIFIC study, and this approach has become the standard care of locally advanced NSCLC (298). Clinical trials evaluating the concurrent administration of radiation therapy with ICIs are ongoing.
3. Vaccines using cancer-specific peptides or DCs (237, 299, 300) and oncolytic virus therapy (301, 302) escalate the antigen presentation and priming of T cells.

### Target Oncogenic Genes

1. Blocking the MAPK/PTEN/PI3K axis such as BRAF, MEK, and PI3K inhibitors contributes to Teff expansion, avoiding T-cell exhaustion and apoptosis, activating an immune-stimulatory transcriptional program, and promoting the production of proinflammatory cytokines and T-cell cytotoxicity (303, 304). BRAF and MEK inhibitors in combination with PD-1 blockade therapy showed a 73% overall response rate (ORR) and 93% stable disease in BRAF V600-mutated metastatic melanoma (305). However, cobimetinib, a MEK1/2 inhibitor, combined with atezolizumab was evaluated in a phase Ib umbrella platform MORPHEUS. The combination did not show better efficacy compared with the control arm in the NSCLC cohort of this study (306).
2. PARP inhibitors, as synergistic activating CD8+T-cell-mediated antitumor response despite up-regulating PD-L1 expression, which can be complementally inhibited by anti-PD-L1 therapy (307).
3. The combinations of nivolumab with veliparib and pembrolizumab with olaparib were tested in advanced solid tumors (308, 309). No response was observed and PFS and OS were 9.0 and 26.8 weeks, respectively, in the former trial while no results are available in the latter one.

### Promote T-Cell Priming and Enhance TILs

(1) Agonists of TLRs, as contributing to the DC mutations and T-cell priming; (2) STING, as activating inflammatory reactions via IFN-α cascade upon recognition of foreign DNA; (3) dual block CTLA-4 and PD-1, as CTLA-4 inhibitor enhances the T-cell priming, Treg exhaustion, and CTL-mediated immune responses via more antigen recognition (310), while PD-1 inhibitor participates in later reactivation of Teff response; (4) adoptive T-cell transfer either alone or in combination with ICIs therapy, as increasing TILs and T-cell cytotoxicity (311, 312); and (5) bispecific monoclonal antibodies, as redirecting cytotoxic effector cells to the TME, depleting suppressive cells, and activating effector cells by targeting a cancer-specific antigen and either CD3 on CTLs or CD16A on NK cells; or targeting cancer-specific antigen and immune regulators, or targeting dual immunomodulators (313).

### Reshape Immunosuppressive TME

(1) Colony-stimulating factor 1 receptor (CSF1R) blockades, as reducing tumor invasion via the MDSCs and M2 macrophages; (2) inhibition of CD73, A2A receptor, as improving TME by targeting suppressive factors; (3) dual blockade of the TGF-β and checkpoint inhibitory receptors, as facilitating tumor penetration with T cells and reversing the immune suppressive TME (208); (4) anti-CXCR2/CXCR4 antibodies, as voiding immune evasion (314); (5) VEGF inhibitors, as normalizing the immune suppressive TME and reversing ICIs resistance (315); and (6) IL-1β inhibitor canakinumab, as targeting tumor inflammatory response and reducing immunosuppression. To date, there are four clinical trials of canakinumab in various settings in the treatment of NSCLC under way, and preliminary results from two of them were released in AACR this year. Pembrolizumab plus chemotherapy combined with canakinumab was safe and well tolerated in the first-line treatment in locally advanced or advanced NSCLC, and the recommended phase III dose of canakinumab was 200 mg s.c. Q3W (316). The efficacy of canakinumab or pembrolizumab monotherapy or in combination as neoadjuvant treatment in resectable NSCLC was assessed in the CANOPY-N study and the results are not reported (317).

### Target Alternate Immune Checkpoints and Immune-Stimulatory Receptors

(1) Blockade of alternate coinhibitory immune checkpoint receptors, such as LAG-3, TIM-3, TIGIT, BTLA, VISTA, and SIGLEC9; (2) costimulatory agonists, including 4-1BB, OX40, CD40, GITR, and ICOS, as enhancing T-cell expansion and effector functions while controlling Treg cell-suppressive functions (318, 319).

Although not being widely used in clinical practice, the antibodies targeting these immune checkpoints have exhibited promising antitumor activity in early clinical trials in various malignancies. LAG-3 blockades, Relatlimab [humanized anti-LAG-3 monoclonal antibody (mAb)], and Eftilagimod alpha (a soluble LAG-3 protein) combined with PD-1 inhibitors achieved an ORR of 15% in previously treated melanoma (320) and 52.9% in treatment-naïve advanced NSCLC (321), respectively. Based on the positive preclinical results, TIGIT blockade, especially in combination with anti-PD-1/PD-L1 mAb, has been explored in various clinical settings of advanced tumors. MK-7684 (an anti-TIGIT mAb) alone or combined with pembrolizumab showed a disease control rate of 35% and 47%, respectively, in a phase I study (322). Etigilimab (a humanized anti-TIGIT mAb) also presented early signs of efficacy as a monotherapy, with a 0% ORR but 22% stabilized disease in advanced malignancies (322). Tiragolumab is a fully human IgG1/kappa TIGIT monoclonal antibody with an intact Fc region that blocks TIGIT from binding to its PVR ligand and to the co-activating receptor CD226. It improved ORR either alone or combined with atezolizumab compared to historical data in a phase I study (323). The clinically meaningful improvement in ORR and PFS was confirmed recently in the CITYSCAPE study (a phase II study of tiragolumab plus atezolizumab vs placebo plus atezolizumab as first-line treatment in patients with PD-L1-selected NSCLC) (324). Cobolimab is a novel IgG4 anti-TIM-3 mAb and showed clinical benefit with an ORR of 15% and 40% stable disease in combination with anti-PD-1 mAb in a phase I clinical trial (325). Other anti-TIM-3 mAbs including MBG453, Sym023, INCAGN2390, LY3321367, BMS-986258, and SHR1702, as well as a bispecific antibody targeting PD-1 and TIM-3 (RO7121661), have also being evaluated in phase I trials with no clinical results available.

Apart from blocking coinhibitory immune checkpoint receptors mentioned above, several costimulatory agonists are also attractive targets, a few of which have stepped into clinical studies. ATOR-1015, a CTLA-4 × OX40 bispecific antibody, was tested in a phase I study for safety and tolerability in advanced solid tumors (326). GSK998, a humanized IgG1 agonistic OX40 mAb, combined with or without pembrolizumab was also evaluated in a phase I trial of advanced solid tumors including NSCLC (327). A lipid nanoparticle encapsulated mRNA encoding human OX40L, mRNA-2416, showed good tolerability when intratumorally injected as monotherapy in advanced malignancies in a phase I/II trial, and the combination with durvalumab is ongoing (328).

### Epigenetic Modulation

(1) DNA methyltransferase inhibitors, e.g., sensitizing tumors to PD-L1 blockade and elevating the secretion of the immunostimulatory chemokines CXCL10 and CXCL9 (155); (2) histone deacetylase inhibitors, as down-regulating MDSCs, increasing the expression of MHC-I and antigen presentation, and increasing tumor-infiltrating CD8^+^ T cells (95); and (3) histone methyltransferase Ezh2 inhibitor, as reversing the effects of loss of immunogenicity and antigen presentation (94).

### Gut Microbiota Modulation

Modifying the composition of gut microbiome might eliminate resistance to ICIs. Dietary modification, probiotics, and fecal microbiota transplantation have been emerging as an adjunct treatment to ICIs.

Of note, combination strategies that have been successful in preclinical models do not necessarily pass safety and performance assessments in clinical trials. In addition to considering the complementarity of immunotherapy resistance mechanisms, the timing and sequence are also important when formulating combination treatment strategies. Therefore, the preclinical model, translational study, and pharmacokinetic study of each of these agents in combination and in isolation are indispensable for the clinical success of combination strategies. Furthermore, multimodal approaches, for example, local therapy for oligo-progression after response to ICIs, should be implemented on therapeutic combinations for better clinical benefits.

## Conclusion

It remains challenging to clarify the resistance mechanisms of immunotherapy since they are complex and dynamic, and certain mechanisms alternately overlap. Further understanding of the primary and acquired resistance mechanisms of immunotherapy will help clinicians to make reasonable combination treatment decisions to bring superior survival and avoid additional toxicity for patients with lung cancer.

## Author Contributions

FW conceptualized and drafted the review. QZ and SW contributed to the significant portions of the manuscript. All authors listed approved it for publication.

## Conflict of Interest

The authors declare that the research was conducted in the absence of any commercial or financial relationships that could be construed as a potential conflict of interest.

## Supplementary Material

Supplementary Table 1 Combination therapeutic strategies and examples of ongoing clinical trials to overcome ICIs resistance in advanced lung cancer.
