# Supplementary material for: The Resistance Mechanisms of Lung Cancer Immunotherapy
Source: Front Oncol. 2020 Oct 20;10:568059. doi: 10.3389/fonc.2020.568059 (PMC7606919; doi:10.3389/fonc.2020.568059)
Supplement: Supplementary Table 1 — Combination therapeutic strategies and examples of ongoing clinical trials to overcome ICIs resistance in advanced lung cancer. [file Table_1.docx]

**Supplementary Table 1: Combination therapeutic strategies and examples of ongoing clinical trials to overcome ICIs resistance in advanced lung cancer**

| Combination approach | Mechanism of action | Combination agents | Phase | Clinical trials number |
| --- | --- | --- | --- | --- |
| Chemotherapy | Increasing immunogenic tumor cells death; promoting antigen presentation and T cells priming; depleting MDSCs and Tregs. | Durvalumab + tremelimumab vs. Durvalumab + chemotherapy vs. chemotherapy | 3 | NCT03164616  (POSEIDON) |
| Radiation therapy | Increasing immunogenic tumor cells death; promoting antigen presentation and pro-inflammatory cytokines release; activating DCs. | Pembrolizumab + SBRT vs. placebo + SBRT | 3 | NCT03924869  (Keynote 867) |
| Immune checkpoint inhibitors | Promoting T cells priming, enhancing T cells cytotoxic function; reversing T cells exhaustion; widening TCR repertoire; and depleting Tregs. | Pembrolizumab +ipilimumab vs. pembrolizumab | 3 | NCT03302234 (Keynote 589) |
|  |  | Pembrolizumab + eftilagimod alpha (LAG-3) | 2 | NCT03625323 |
|  |  | Atezolizumab + tiragolumab (TIGIT) vs. atezolizumab | 2 | NCT02794571 |
|  |  | Pembrolizumab + enoblituzumab (B7-H3) | 1 | NCT02475213 |
|  |  | Nivolumab + BMS-986258 (TIM3) | 1 | NCT03446040 |
| Costimulatory agonists | Enhancing T cells cytotoxic function; intensifying T cells activation; improving T cells survival and memory formation. | INBRX-106 (OX40) ± pembrolizumab | 1 | NCT04198766 |
|  |  | APX005M (CD40) + nivolumab | 1/2 | NCT03123783 |
|  |  | INCAGN01876 (GITR) + nivolumab + ipilimumab | 1/2 | NCT03126110 |
|  |  | Vopratelimab (ICOS) + ipilimumab | 2 | NCT03989362 |
| Targeting cytokine/chemokine | Enhancing T cells cytotoxic function; improving T cells infiltration; preventing immunosuppressive cells recruitment; inhibiting tumor invasion and metastasis; reversing immunosuppressive TME. | Vactosertib (TGF-β) + pembrolizumab | 2 | NCT04515979 |
|  |  | Canakinumab (IL-1β)+ pembrolizumab + chemotherapy vs. pembrolizumab + chemotherapy | 3 | NCT03631199 |
|  |  | CAN04 (IL1RAP) + pembrolizumab | 1 | NCT04452214 |
|  |  | Certolizumab (TNF-α) + chemotherapy | 1 | NCT02120807 |
|  |  | BMS-813160 (CCR2/5) + BMS-986253 (IL-8) + nivolumab | 1 | NCT04123379 |
|  |  | Cabiralizumab (CSF1R) + APX005M + nivolumab | 1 | NCT03502330 |
| Bispecific antibodies | Amplifying costimulation of immune cells; activating and redirecting cytotoxic T cells; depleting immunosuppressive cells. | M7824 (PD-1/TGF-β) vs. pembrolizumab | 3 | NCT03631706 |
|  |  | RO7247669 (PD-1/LAG3) | 1 | NCT04140500 |
|  |  | RO7121661 (PD-1/TIM-3) | 1 | NCT03708328 |
|  |  | INBRX-105 (PD-L1/41BB) | 1 | NCT03809624 |
|  |  | ES101 (PD-L1/41BB) | 1 | NCT04009460 |
|  |  | AMG757 (DLL3/CD3) ± pembrolizumab | 1 | NCT03319940 |
|  |  | CDX-527 (PD-L1/CD27) | 1 | NCT04440943 |
|  |  | XmAb®22841 (CTLA4/LAG3) ± pembrolizumab | 1 | NCT03849469 |
|  |  | BCA101 (EGFR/TGF-β) ± pembrolizumab | 1 | NCT04429542 |
|  |  | CC-1 (PSMA/CD3) + toczilizumab (IL-6) | 1/2 | NCT04496674 |
| TLR agonists | Boosting DCs maturation and T cells priming; inducing inflammatory cytokines release. | BNT411 ± atezolizumab, chemotherapy | 1/2 | NCT04101357 |
| Targeted therapy | Blocking oncogenic signaling pathways; increasing genomic instability; improving T cells proliferation; promoting T cells infiltration and cytotoxic function; reversing T cells exhaustion; reducing immunosuppressive cytokines production; normalizing TME; modulating metabolic response. | Cobimetinib (MEK) + atezolizumab | 2 | NCT03600701 |
|  |  | Idelalisib (PI3K) + pembrolizumab | 1/2 | NCT03257722 |
|  |  | Bevacizumab + chemotherapy + atezolizumab vs. bevacizumab + chemotherapy | 3 | NCT04194203 (IMPOWER151) |
|  |  | AB928 (A2AR) + AB154 (TIGIT) + zimberelimab | 2 | NCT04262856 |
|  |  | Pembrolizumab + olaparib vs. pembrolizumab + pemetrexed | 3 | NCT03976323  (KEYLYNK006) |
|  |  | Avelumab + axitinib + palbociclib | 1/2 | NCT03386929 |
|  |  | Durvalumab + sirolimus | 1 | NCT04348292 |
|  |  | Trastuzumab deruxtecan + pembrolizumab | 1 | NCT04042701 |
|  |  | Capmatinib + pembrolizumab vs pembrolizumab | 2 | NCT04139317 |
| STING agonists | Evoking INF-α signaling pathway; activating JAK/STAT signaling pathway; promoting DCs function and T cells priming. | Danvatirsen + durvalumab | 1 | NCT02983578 |
| Epigenetic modulators | Altering immune-related genes expression; promoting antigen presentation and immune-stimulatory cytokines release | Decitabine + tetrahydrouridine (hypomethylating agents) + pembrolizumab | 1/2 | NCT03233724 |
|  |  | Entinostat (histone deacetylase inhibitor) + pembrolizumab | 1/2 | NCT02437136 |
| ACT therapy | Targeting tumor-specific antigens; improving T cells infiltration and cytotoxicity; inducing inflamed TME. | Anti-MUC1 CAR T cells + PD-1 knockout engineered T cells | 1/2 | NCT03525782 |
| Oncolytic virus | Increasing immunogenic tumor cells death; improving antigen presentation; boosting T cells priming and DCs function. | ADV/HSV-tk + valacyclovir + SBRT + pembrolizumab | 2 | NCT03004183 |
| Cancer vaccine | Improving antigen presentation;  Increasing tumor-specific T cells; enhancing T cells infiltration. | Nivolumab + ipilimumab + DC based p53 vaccine | 2 | NCT03406715 |
| Gut microbiome modulators | Normalizing immunosuppressive TME; inducing inflammatory cytokines release; improving host immune status. | Nivolumab + fecal microbiota transplantation | 1/2 | NCT04521075 |

Abbreviations: MDSCs: myeloid derived suppressor cells; Tregs: regulatory T cells; DC: dendritic cells; TCR: T-cell receptor; TLR: Toll-like receptor; ACT: adoptive T cell; SBRT: stereotactic body radiation therapy; TME: tumor microenvironment; IL-1RAP: interleukin-1 receptor accessory protein; CAR: chimeric antigen receptor; ADV/HSV-tk: adenovirus-mediated expression of herpes simplex virus thymidine kinase.
